# Pt(II) and Pd(II) Complexes with *β*-Alanine

**DOI:** 10.1155/2008/983725

**Published:** 2008-05-26

**Authors:** L. F. Krylova, L. M. Kovtunova, G. V. Romanenko

**Affiliations:** ^1^Novosibirsk State University, 2 Pirogova Street, Novosibirsk 630090, Russia; ^2^International Tomography Center, Siberian Branch of the Russian Academy of Sciences, 3a Institutskaya Street, Novosibirsk 630090, Russia

## Abstract

A sequence of stages in the syntheses of isomeric bisamino acid complexes of Pt(II) with *β*-aminopropionic acid (*β*-alanine = *β*-AlaH) has been studied by the ^195^Pt NMR spectroscopy. The techniques have been developed of the synthesis of the *cis*- and *trans*-bischelates of Pt(II) and Pd(II) with *β*-alanine as well as of the halide complexes of *trans*-[M(*β*-AlaH)_2_Cl_2_] (M = Pt, Pd) and *trans*-K_2_[Pt(*β*-Ala)_2_I_2_] types. The NMR spectroscopy and IR spectroscopy (in the nuclei of ^195^Pt,^13^C,^1^H) and X-ray diffraction analysis have been used to examine the structures of the synthesized compounds.

## 1. INTRODUCTION

The neoplastic activity of some Pt(II) complexes was discovered in 1971 [1]. It has given an impulse to the growth of platinum metal complexation. The study of the Pt(II) and Pd(II) complexes with amino acids is very promising due to both of their biological activity and their use in the solution of the fundamental problems in coordination chemistry of planar complexes with multifunctional ligands. The Pt(II) and Pd(II) complexes with *α*-alanine (NH_2_CH(CH_3_)COOH) are widely described in the literature [2–8].

Less attention has been paid to the Pt(II) complexes with the simplest *β*-amino acid (*β*-alanine, *β*-AlaH– NH_2_(CH_2_)_2_COOH). The syntheses of two bisaminoacid complexes are described in [9]: those of *trans*-[Pt(*β*-Ala)_2_] where *β*-alanine is a bidentate ligand (coordinated via the NH_2_ and OCO groups) and of *trans*-[Pt(*β*-AlaH)_2_Cl_2_] where *β*-alanine is a monodentate ligand (coordinated via the NH_2_ group). The *trans*-configuration of these complexes has been proved by chemical methods. No physical methods have been applied to verify their structures.

The monoaminoacid complex K[Pt(NO_2_)_2_(*β*-Ala)] with the bidentate *β*-alanine as well as the bisaminoacid complexes [Pt(NO_2_)_2_(*β*-AlaH)_2_] and K_2_[Pt(NO_2_)_2_(*β*-Ala)_2_] with the monodentate *β*-alanine were described in [10]. On the basis of the Kurnakov reaction (thiourea test), it was concluded [10] that the bisaminoacid complexes of Pt(II) appear to have the *trans*-configuration. The Cambridge crystallographic database only contains some information about one Pt(II) complex with *β*-AlaH, [Pt(*β*-Ala)(C_2_H_4_)Cl], where *β*-Ala has a bidentate coordination to the Pt atom [11]. So far there has been no evidence of *β*-AlaH bisaminoacid complexes of Pt(II) with a *cis-*arrangement of ligands. To the authors knowledge the Pd(II) complexes with *β*-AlaH have never been investigated either.

In this work, the ^195^Pt NMR spectroscopy has been used to study the successive stages of the synthesis of bisaminoacid Pt(II) complexes with *β*-alanine, which can exist in *trans-* and *cis-*configurations. On the basis of the data obtained, we have developed the methods of synthesizing different Pt(II) and Pd(II) bisaminoacid complexes with *β*-alanine. The complexes in question have been characterized by ^1^H, ^13^C NMR, IR spectroscopy, and X-ray diffraction analysis.

## 2. EXPERIMENTAL

### 2.1. Syntheses of the *trans*-[Pt(*β*-AlaH)_**2**_Cl_**2**_] and *trans*-[Pt(*β*-Ala)_**2**_] complexes

The *trans*-[Pt(*β*-AlaH)_2_Cl_2_] and *trans*-[Pt(*β*-Ala)_2_] complexes were synthesized using the techniques suggested in [9], which were slightly modified for the purpose (see Section 3).

### 2.2. Synthesis of the *trans*-[Pd(*β*-AlaH)_**2**_Cl_**2**_] complex

0.887 g (5 mmol) of PdCl_2_ and 0.585 g (10 mmol) of NaCl were dissolved in 20 mL of water and heated in the water bath until the dilution of PdCl_2_. A solution containing 1.78 g (20 mmol) of *β*-AlaH and 0.800 g (20 mmol) of NaOH in 20 mL of H_2_O was added to the solution of K_2_[PdCl_4_]. After cooling the solution as low as 0°C, 10 mL of concentrated HCl was added to the reaction mixture. The orange precipitate fell out, which was filtered off in an hour, washed with cold water, and dried at room temperature. The yield was up to 85%.

Anal. found (%); C 19.7, H 3.93, N 8.02, Cl 19.5, Pd 29.5. Calcd. for C_6_H_14_N_2_O_4_Cl_2_Pd (%): C 20.3, H 3.94, N 7.88, Cl 20.0, Pd 29.9.

### 2.3. Synthesis of the *trans*-[Pd(*β*-Ala)_**2**_] complex

1.0 g of the *trans*-[Pd(*β*-AlaH)_2_Cl_2_] was dissolved in 10 mL of water then the reaction mixture was treated with 1 M of NaOH till it became neutral to phenolphthaleine. A light yellow precipitate fell out, which was filtered off and washed with cold water. At first it was dried at room temperature and then at 110°C. The yield was up to 76%.

Anal. found (%); C 25.5, H 4.17, N 9.88, Pd 37.9. Calcd. for C_6_H_12_N_2_O_4_Pd (%): C 25.5, H 4.25, N 9.92, Pd 37.7.

### 2.4. Synthesis of the *cis-*[Pd(*β*-Ala)_**2**_] complex

0.5 g of the *trans*-[Pd(*β*-Ala)_2_] was dissolved in 20 mL of water. The reaction mixture was heated with stirring at 80°C for 3 hours. After cooling the reaction mixture as low as 0°C, a small amount of the starting *trans*-bischelate was filtered off. Then the *cis*-[Pd(*β*-Ala)_2_] was settled with acetone from the filtrate solution (water : acetone ~ 1 : 4) and was filtered off. The solid *cis*-[Pd(*β*-Ala)_2_] was dried at room temperature at first and then at 110°C. The yield was up to 60%.

Anal. found (%); C 25.0; H 4.30; N 10.0; Calcd. for C_6_H_12_N_2_O_4_Pd (%): C 25.5; H 4.25; N 9.92.

### 2.5. Synthesis of the *cis*-[Pt(*β*-Ala)_**2**_] complex

3.32 g (20 mmol) of KI in 25 mL of water was added to 2.08 g (5 mmol) of K_2_[PtCl_4_] in 25 mL of water. The reaction mixture was heated in the water bath for 10 minutes. A solution of *β*-AlaH (0.89 g, 10 mmol) in water (25 mL) was added to the reaction mixture, which was heated in the water bath for 2 hours. While heating the reaction mixture, 10 mL of 0.5 M of KOH was added by small portions. Then a small amount of black precipitate was filtered off. The solution of AgNO_3_ (3.40 g, 20 mmol) in water (25 mL) was added to the orange solution of the filtrate obtained, and the mixture was heated for ~5 minutes. The coagulated AgI precipitate was filtered off. The reaction product was precipitated with acetone (~100 mL) from the filtrate (V ~50 mL). The yield was up to 20%.

Anal. found (%): Pt 53.1, N 7.65; Calcd. for C_6_H_12_N_2_O_4_Pt (%): Pt 52.6, N 7.55.

### 2.6. Measurements

*NMR spectra* were recorded using a Bruker DPX-250 spectrometer at the frequencies of 250 (^1^H), 62.9 (^13^C), and 53.6 (^195^Pt) MHz. Two solvents, D_2_O and acetone-d_6_, were used for the ^1^H NMR spectrum: (a) in the D_2_O solution, the chemical shifts were determined with reference to the signal of the CH_3_-group protons of the DMSO, which was added as an internal standard (*δ* = 2.660 ppm); (b) in the acetone-d_6_ solution, the chemical shifts were determined with reference to the central signal of the acetone residual protons (*δ* = 2.070 ppm). For the ^13^C NMR spectrum the same solvents, D_2_O and acetone-d_6_, were used: (a) in the D_2_O solution the chemical shifts were determined with reference to the ^13^C signal of the DMSO, which was added as an internal standard (*δ* = 40.2 ppm); (b) in the acetone-d_6_ solution the chemical shifts were determined with reference to the signal of the methyl carbon atom of acetone-d_6_ (*δ* = 29.2 ppm). The chemical shift of the ^195^Pt NMR signals was recorded with regard to the external standard, that is, 1 M of the Na_2_[PtCl_6_] water solution. All measurements were performed at room temperature. The ^195^Pt and ^13^C NMR spectra were recorded using proton decoupling.

*The IR spectra* of crystalline samples packed in the KBr pellets were measured using a Bruker Vector-22 one-beam FT spectrophotometer.

*X-ray diffraction analysis*. Single-crystal data were collected on a SMART APEX CCD (Bruker AXS) diffractometer (Mo K*α*, *λ* = 0.71073 Å, *T* = 298 K, an absorption correction applied using the Bruker SADABS program, version 2.10). The structures were solved by the direct methods and refined by the full-matrix least squares in an anisotropic approximation for all nonhydrogen atoms. The H atoms were located in difference electron density syntheses and refined together with nonhydrogen atoms in an isotropic approximation. All calculations on the structure solution and refinement were carried out with the Bruker Shelxtl Version 6.14 software.

**Trans-[Pd(*β*-Ala)_2_]**

C_6_H_12_N_2_O_4_Pd, *M* = 282.58, monoclinic crystals, space group *P*2_1_/n, *a* = 5.750(1) Å, *b* = 8.910(2) Å, *c* = 9.020(2) Å, *β* = 104.692(3)°, *V* = 447.0(2) Å^3^; *Z* = 2, *ρ*_calc_ = 2.100 g · cm^−3^, *μ* = 2.061 mm^−1^, 3281 reflections collected (3.27 < *θ* < 23.28°, *R*_int_ = 0.0242), including 639 reflections with *I* > 2*σ*(*I*), 86 refined parameters, *R*_1_ = 0.0156, *w* *R*_2_ = 0.0634.

**Trans-[Pt(*β*-Ala)_2_]**

C_6_H_12_N_2_O_4_Pt, *M* = 371.26, monoclinic crystals, space group *P*2_1_/*c*, *a* = 9.197(4) Å, *b* = 11.080(5) Å, *c* = 8.656(4) Å, *β* = 98.474(6)°, *V* = 872.4(7) Å^3^; *Z* = 4, *ρ*_calc_= 2.827 g · cm^−3^, *μ* = 160.7 cm^−1^, 6471 reflections collected (3.53 < *θ* < 23.33°, *R*_int_ = 0.0388), including 1253 reflections with *I* > 2*σ*(*I*), 134 refined parameters, *R*_1_ = 0.0290, *w* *R*_2_ = 0.0618.

**Cis-[Pt(*β*-Ala)_2_]**

C_6_H_12_N_2_O_4_Pt, *M* = 371.26, monoclinic crystals, space group *P*2_1_/*c*, *a* = 17.673(3) Å, *b* = 10.232(3) Å, *c* = 10.457(2) Å, *β* = 100.043(4)°, *V* = 1862.0(6) Å^3^; *Z* = 8, *ρ*_calc_ = 2.649 g · cm^−3^, *μ* = 150.6 cm^−1^, 6570 reflections collected (2.90 < *θ* < 23.33°, *R*_int_ = 0.0673), including 2608 reflections with *I* > 2*σ*(*I*), 258 refined parameters, *R*_1_ = 0.0381, *w* *R*_2_ = 0.0824.

**Trans-K_2_[Pt(*β*-Ala)_2_I_2_]^•^2H_2_O**

C_6_H_16_I_2_K_2_N_2_O_6_Pt, *M* = 739.30, monoclinic crystals, space group *P*2_1_/*c*; *a* = 5.0414(7) Å, *b* = 24.174(3) Å, *c* = 7.0414(10) Å, *β* = 94.045(2)°, *V* = 856.0(2) Å^3^, *Z* = 2, *ρ*_calc_ = 2.868 g · cm^−3^, *μ* = 123.1 cm^−1^, 6375 reflections collected (1.68 < *θ* < 23.29°, *R*_int_ = 0.0318), including 1231 reflections with *I* > 2*σ*(*I*), 121 refined parameters, *R*_1_ = 0.0203, *w* *R*_2_ = 0.0475.

## 3. RESULTS AND DISCUSSION

### 3.1. Synthesis of the Pt(II) *trans*-isomers (Scheme 1)

The solutions of *β*-AlaH neutralized with an alkali and K_2_[PtCl_4_] were used as the reagents for the synthesis of *trans*-isomers. The molar ratio of the reagents K_2_[PtCl_4_] : *β*-AlaH : KOH was as follows 1 : 4.5 : 2. The structures of complexes were detected by the ^195^Pt NMR spectroscopy at each stage of the synthesis.

**Scheme 1:**
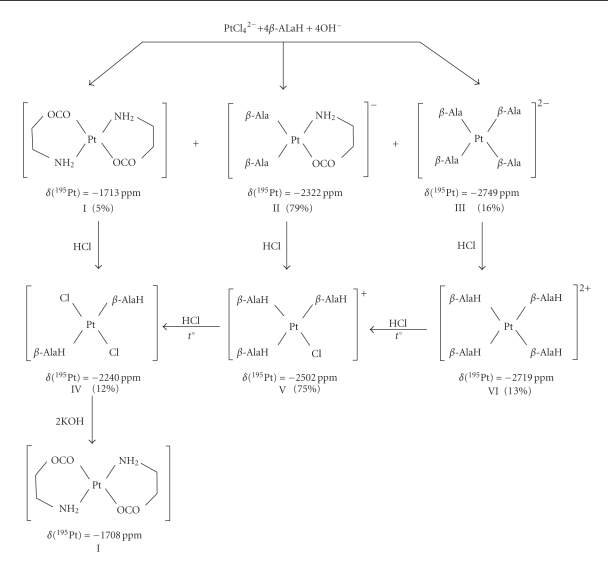
Sequence of reactions of K_2_[PtCl_4_] with *β*-alanine.

According to the data of [9], the first stage of the synthesis is heating of the reaction mixture for 5 hours. We have shown that after heating the aqueous solution of K_2_[PtCl_4_] with the neutralized *β*-AlaH for 2 hours, the reaction mixture does not contain the starting reagents. Instead, it contains three forms (I, II, and III) of the Pt(II) complexes (Scheme 1), complex II being predominant (~80%). The signal assignment in the ^195^Pt NMR spectra was carried out using the data of [12].

The second stage comprises the interaction of complexes with HCl. Just after the addition of HCl to the reaction mixture, it is detected that the solution contains complexes IV, V, and VI, complex V prevailing (~75%).

Complex IV is formed from complex I as a result of the ring opening and the insertion of Cl^−^ ions at the site of cleavage of the Pt–OCO bond. Complex V is formed from complex II via the ring opening and the insertion of the Cl^−^ ion. Complex VI is formed from complex III via the protonation of the *β*-alaninate ions of complex III. The subsequent heating of the reaction mixture in the water bath for ~20 minutes only results in one complex (complex IV) in the solution. During this period, complex VI converts into complex V, while complex V transforms into the *trans-*dichloride IV due to the replacement of *β*-AlaH with the Cl^−^ ion on the coordinate Cl-Pt-*β*-AlaH. The given replacement takes place in accordance with the kinetic effect of the *trans-*influence of ligands. That is, the *trans*-effect of Cl exceeds that of the NH_2_, the group of *β*-alanine [13].

At the next stage, the yellow precipitate of the *trans*-[Pt(*β*-AlaH)_2_Cl_2_](IV) gradually settles from the solution. The titration of complex IV with the KOH solution leads to the formation of yellow K_2_[Pt(*β*-Ala)_2_Cl_2_] solution, which is then heated to form a white precipitate of *trans-*[Pt(*β*-Ala)_2_](I).

### 3.2. Synthesis of *trans*-isomers of the Pd(II) complexes

The problems of isolation of the individual geometrical isomers of the Pd(II) bisaminoacid complexes with *α*-amino acids are related to the *trans-cis* isomerization processes. For the first time we described these processes for Pd(II) bischelates with glycine and *α*-alanine in [5, 14].

The decrease of temperature from 100°C to 0°C at the appropriate stages of the synthesis rules out the isomerization processes and allows us to use the same approaches to the synthesis of the Pd(II) complexes as is the case with the Pt(II) complexes.

The interaction of Na_2_[PdCl_4_] with the neutralized *β*-AlaH in an aqueous solution is likely to result in the formation of complexes similar to the complexes I, II, and III (Scheme 1). The treatment with HCl_conc_ leads to the *trans-*[Pd(*β*-AlaH)_2_Cl_2_] as the only product. The formation of a *trans*-isomer as the only product is possible due to the kinetic effect of the *trans-*influence of ligands as is the case with the Pt complexes [13].

The titration of the *trans-*[Pd(*β*-AlaH)_2_Cl_2_] with an alkaline solution leads to the ring closure and the formation of the *trans-*[Pd(*β*-Ala)_2_]. The ring closure reaction was conducted at room temperature because the *trans*-bischelate product precipitated immediately. As the *cis*-bischelate readily dissolves in water, it cannot be present in the solid precipitate of the *trans-*[Pd(*β*-Ala)_2_] as an impurity.

The treatment of the *trans*-[Pd(*β*-Ala)_2_] with HCl at ~0°C leads to the opening of amino acid cycles and the formation of the *trans*-[Pd(*β*-AlaH)_2_Cl_2_].

### 3.3. Synthesis of the *cis*-[Pd(*β*-Ala)_**2**_] complex

Due to its high solubility in water, the *cis-*[Pd(*β*-Ala)_2_] complex can hardly be isolated as a solid. That is why the procedures that we have developed for the synthesis of the Pd(II) *cis-*bischelates with valine [15] are not applicable for the preparation of the solid *cis*-[Pd(*β*-Ala)_2_] phase. The kinetic and thermodynamic data for the *trans-cis* isomerization of the Pd(II) bischelates with valine are reported in [15]. It can be supposed that the equilibrium and rate constants of the isomerization reaction are almost similar for the bischelates where amino acids are bonded to Pd(II) via the NH_2_ or OCO groups. Thus for the synthesis of the *cis-*[Pd(*β*-Ala)_2_], we have used the kinetic and thermodynamic data of [15].

The *trans*-[Pd(*β*-Ala)_2_] was heated with water at 80°C until the starting precipitate was completely diluted. The *trans*-isomer isomerized, and the *cis*-isomer stayed in solution. At 80°C the equilibrium constant of the *trans-cis* process was lower than at low temperatures because the reaction is exothermal (see [15]). So the reaction mixture was abruptly cooled to ~0°C in order to increase the concentration of the *cis*-bischelate. Moreover after cooling, the starting *trans*-bischelate, which did not isomerize, precipitated and was filtered off. The solid *cis-*[Pd(*β*-Ala)_2_] was settled with acetone from the aqueous filtrate solution.

The treatment of the *cis-*[Pd(*β*-Ala)_2_] with HCl did not allow us to form the *cis-*[Pd(*β*-AlaH)_2_Cl_2_] as is the case with the *trans*-isomers. Even a highly diluted solution of HCl resulted not only in the opening of the amino acid cycles, but also in completing the substitution of the amino acid ligands and the formation of PdCI_4_^2−^. Similar processes were observed for the Pd(II) complexes with valine [15] and for the Pd(II) complexes with aminobutyric acid [16].

### 3.4. Synthesis of the *cis*-isomers of the Pt(II) complexes (Scheme 2)

For the synthesis of the *cis*-bischelate, we have used K_2_[PtI_4_], which is formed by the reaction of K_2_[PtCl_4_] with KI.

We supposed that the heating of K_2_[PtI_4_] with *β*-AlaH at pH ~6-7 (pH was kept at this level by adding KOH) led to the formation of *cis*-K_2_[Pt(*β*-Ala)_2_I_2_]. The formation of the *cis*-isomer was expected to be in agreement with the kinetic effect of the *trans-*influence (TI) of the ligands (TI (I^−^) ≫ TI(NH_2_)).

Further heating of the *cis-*K_2_[Pt(*β*-Ala)_2_I_2_] does not lead to the ring closure as is the case with the *trans*-dichlorides. Instead, it leads to the isomerization of the *cis-*K_2_[Pt(*β*-Ala)_2_I_2_] and the formation of the *trans*-K_2_[Pt(*β*-Ala)_2_I_2_]. After heating for 10 hours, the solution only contains one form of *δ*(^195^Pt) = −3408 ppm. This new form was isolated as a single crystal and identified by the X-ray diffraction analysis, which confirmed that it was the *trans-*K_2_[Pt(*β*-Ala)_2_I_2_].

To synthesize the *cis*-[Pt(*β*-Ala)_2_], a solution of AgNO_3_ was added to the solution of the *cis*-K_2_[Pt(*β*-Ala)_2_I_2_]. The resulting AgI precipitate was filtered off. The filtrate solution contained only one form of the *cis*-[Pt(*β*-Ala)_2_] complex (*δ*(^195^Pt) = −1923 ppm).

In addition to the main product, the *cis*-[Pt(*β*-Ala)_2_], the solution contained KCl and KNO_3_. We added acetone to the reaction mixture (water : acetone ~ 1 : 2) to separate the desired product from inorganic salts. *Cis*-[Pt(*β*-Ala)_2_] was the only substrate that precipitated under such conditions.

**PMR spectra (Table 1)**

**Figure 1:**
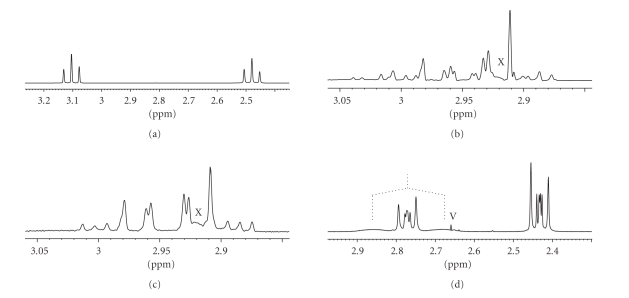
(a) PMR spectra in the region of CH_2_ protons recorded in D_2_O for *β*-AlaH; (b) PMR spectra in the region of CH_2_ protons recorded in acetone-d_6_ for *trans*-[Pt(*β*-AlaH)_2_Cl_2_]; (c) PMR spectra in the region of CH_2_ protons recorded in acetone-d_6_ with NH_2_ proton coupling suppression for *trans*-[Pt(*β*-AlaH)_2_Cl_2_]; (d) PMR spectra in the region of CH_2_ protons recorded in D_2_O for *cis*-[Pt(*β*-Ala)_2_]. X: broadened signal of the H_2_O admixture in acetone (spectra b and c); V: DMSO reference in D_2_O (spectrume d); 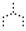: broadened doublet of splitting at ^195^Pt (spectrume d).

The PMR spectrum of *β*-AlaH (Figure 1(a)) in D_2_O contains two triplets of two CH_2_ groups. The spectrum corresponds to an A_2_X_2_ four-spin system with magnetically equivalent protons in each CH_2_ group [17, page 54].

Figure 1 shows that the coordinate *β*-alanine (spectra b, c, and d) has a more complex spectrum than the incoordinate *β*-alanine (spectrum a) in the region of CH_2_ protons. The spectrum contains more lines, and their intensities are distorted. This indicates that the protons of both CH_2_ groups are magnetically nonequivalent. Therefore, these spectra cannot be interpreted using first order rules [17, page 57].

It should be noted that the spectrum of the *trans*-[Pd(*β*-AlaH)_2_Cl_2_] is similar to that of the *trans*-[Pt(*β*-AlaH)_2_Cl_2_]. For both spectra (Figure 1(b)), it is impossible to evaluate the chemical shifts of the individual CH_2_ groups from the spectra. In order to do that we should employ a six-spin system of AA′BB′X_2_ type, where AA′ and BB′ are magnetically nonequivalent protons of the two CH_2_ groups, and X_2_ are magnetically equivalent NH_2_ protons. In this system, the protons of the CH_2_ group, which is related to NH_2_ group, are additionally split at the NH_2_ protons. After the suppression of interaction with the NH_2_ protons, the PMR spectrum (Figure 1(c)) corresponds to an AA′BB′ four-spin system, is symmetric, and allows us to estimate the chemical shifts of the CH_2_ groups on the center of each multiplet [17, page 200].

Figure 1(d) shows the PMR spectrum of the *cis-*[Pt(*β*-Ala)_2_] in D_2_O. The spectrum only contains the signals of two CH_2_ groups (the NH_2_ protons are deuterated in D_2_O and do not display in the spectrum). The PMR spectra of bischelates as well as those of dichlorides allow us to evaluate the chemical shifts of the individual CH_2_ groups on the centers of multiplets.

The spectrum of the *cis-*[Pt(*β*-Ala)_2_] also shows that the weak-field signal of the CH_2_ group combined with the NH_2_ group is split at ^195^Pt (broadened doublet).

It should be noted that for the Pt and Pd *cis*-bischelates we succeeded in recording the signals of the NH_2_ protons in D_2_O because the NH_2_ protons are deuterated in the *trans*-bischelates faster than in the *cis*-bischelates.

**^195^Pt NMR spectra (Table 1)**

The ^195^Pt signal in the spectrum of the *trans-*[Pt(*β*-AlaH)_2_Cl_2_] is found in the region of *δ*⁢ ~ −2200 ppm, as is the case with the other similar compounds with *α*-amino acids. The difference between the chemical shifts of the *cis*- and *trans*-bischelate complexes is up to 200 ppm, the signals of the *trans*-bischelates lying in a weaker field. Such differences are also observed for the bischelates with *α*-amino acids [18].

It should be noted that the signals of *cis-* and *trans-*bischelates with *β*-alanine lie in a stronger field compared to the signals of similar *α*-amino acid complexes, the difference being up to 60–70 ppm.

**^13^C NMR spectra (Table 1)**

The spectrum of the *trans*-[Pt(*β*-Ala)_2_] could not be recorded because of the very low solubility of this compound in water.

Table 1 shows that the ^13^C signals of the protonated COOH group in the *trans-*[M(*β*-AlaH)_2_Cl_2_] (M = Pt, Pd) lie in a stronger field than the signals of the coordinate COO groups in the *cis*- and *trans*-bischelate complexes of Pt(II) and Pd(II).

**IR spectra (Table 2)**

As is known, for free amino acids, which exist as bipolar NH_3_^+^CH(R)COO^−^ ions, there is a broad band of up to 3400 cm^−1^ in the region of the N = H ($\tilde{\nu }$ (NH)) stretching vibrations, while the C = O ($\tilde{\nu }$ (CO)) stretching vibrations display in the region of 1600 cm^−1^.

For the coordinated *α*-amino acids in the Pt(II) and Pd(II) bischelate complexes, the $\tilde{\nu }$ (CO) is recorded in the region of 1650 cm^−1^, while the $\tilde{\nu }$ (NH) is found at 3200 cm^−1^. For example, for the Pt(II) *trans*-bischelate with glycine, the $\tilde{\nu }$ (CO) is equal to 1643 cm^−1^, and the $\tilde{\nu }$ (NH) is equal to 3230 and 3090 cm^−1^. For the similar Pd(II) complex, the $\tilde{\nu }$ (CO) equals 1642 cm^−1^, and the $\tilde{\nu }$ (NH) equals 3230 and 3120 cm^−1^ [19].

For all complexes presented in this work, the split lines of NH antisymmetric stretching vibrations in the region of ~3200 cm^−1^ were found as well as NH symmetric stretching vibrations at ~3100 cm^−1^, that correspond to the coordinated NH_2_ group.

In the region of the C = O stretching vibrations for the Pt(II) and Pd(II) bischelates, the $\tilde{\nu }$ (CO) is in the range of 1617–1640 cm^−1^, which shows that the OCO group is coordinate.

For the *trans*-K_2_[Pt(*β*-Ala)_2_I_2_] complex with the incoordinate COO group, the $\tilde{\nu }$ (CO) is equal to 1602 cm^−1^, as is the case with free amino acids.

For the *trans*-[M(*β*-AlaH)_2_Cl_2_] complexes (where M = Pt, Pd) containing the monodentate ligands of *β*-AlaH, which is coordinated via the NH_2_ group and contains the protonated COOH group, the stretching vibrations, the $\tilde{\nu }$ (C = O), reach up to 1710 cm^−1^, as for similar compounds with *α*-amino acids [19].

**X-ray diffraction data (Table 3)**

**Figure 2:**
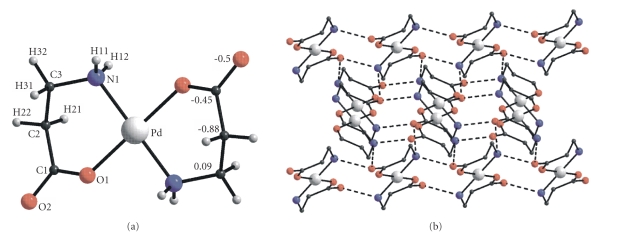
Molecular structure of *trans*-[Pd(*β*-Ala)_2_], atomic numbering, and deviations of atoms (Å) from the plane of the coordination square (a); view of the framework in the structure of complex (b).

In the *trans*-[Pd(*β*-Ala)_2_] centrosymmetric molecule (Figure 2(a)), the Pd–O and Pd–N distances are 2.004 and 2.026 Å. The deviations of atoms from the plane of the chelate ring may be as high as 0.88 Å (for *α*-C). The chelate angle is 94.35°, which corresponds to the transannular distance O⋯N 2.958 Å. The hydrogen bonds between the NH_2_ groups and the incoordinate atoms of carboxyl O groups link the molecules into a framework (Figure 2(b)).

**Figure 3:**
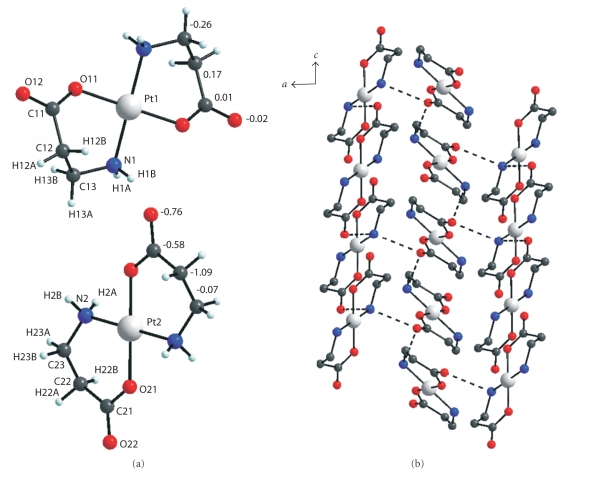
Molecular structure of Pt1 and Pt2 in *trans*-[Pt(*β*-Ala)_2_] (a); structure projected on the (001) plane (b).

In the structure of the *trans*-[Pt(*β*-Ala)_2_], both of the crystallographically independent molecules are also centrosymmetric. The Pt(II) atom is surrounded by a square formed by the N donor atoms of the amino groups and the O atoms of the two alaninate anions (Figure 3(a)). The average Pt–O and Pt–N bond lengths are 1.996 and 2.028 Å, respectively. As in the *trans*-[Pd(*β*-Ala)_2_] , the C–O distances for the coordinate O atom of the deprotonated OH group are appreciably longer than those for the incoordinate atoms (1.29 and 1.23 Å on the average). The independent molecules differ in the configuration of the chelate rings. The maximal deviation of nonhydrogen atoms from the plane of the coordination square is 0.26 Å in the Pt1 molecule and 1.09 Å in the Pt2 molecule (Figure 3(a)). The N–Pt–O chelate angle in the Pt2 molecule, which is “more planar,” is 95.7(3)°; this is much larger than in the Pt1 molecule (94.0(3)°).

The molecules in the structure are hydrogen bonded into a framework by N–H⋯O type bonds (Figure 3(b)).

**Figure 4:**
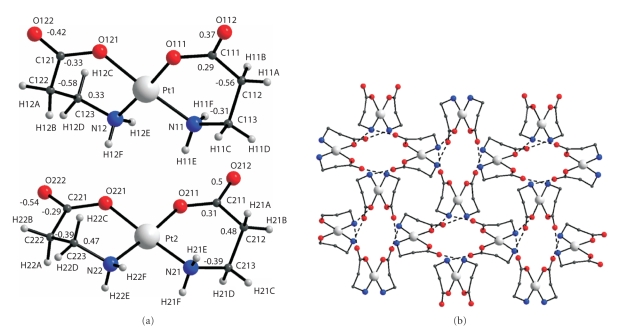
Molecular structure of Pt1 and Pt2 in *cis*-[Pt(*β*-Ala)_2_] and deviations of atoms (Å) from plane of the coordination square (a); structure of a layer (b).

In the crystallographically independent *cis*-[Pt(*β*-Ala)_2_] molecules the metal atom also has square planar surroundings (Figure 4(a)). The average values of the Pt–O and Pt–N bond lengths are 2.015 and 1.997 Å, respectively. The maximal deviation of nonhydrogen atoms from the coordination square plane is 0.58 Å in the Pt1 molecule and 0.54 Å (Figure 4(a)) in the Pt2 molecule. The hydrogen bonds link the molecules into layers (Figure 4(b)).

**Figure 5:**
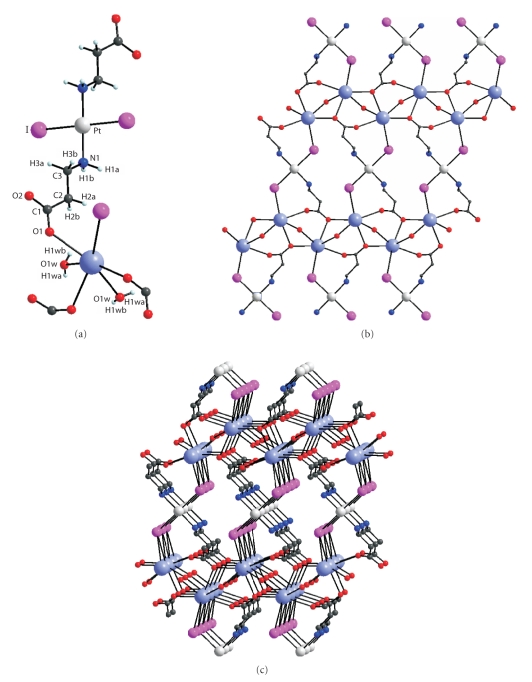
Atomic numbering scheme in an independent fragment (a); structure of the layer (b), and structure of the framework (c) in *trans*-K_2_[Pt(*β*-Ala)_2_I_2_]^•^2H_2_O.

In the *trans*-K_2_[Pt(*β*-Ala)_2_I_2_]^•^2H_2_O complex, the coordination *trans*-square of Pt is formed by two I atoms and by the N atoms of the *β*-alaninate ions. The Pt–I distance is 2.5902(5) Å, and the Pt–N distance is 2.047(5) Å. The N⋯O distance in *β*-Ala is 4.197 Å. The carboxylate groups of the *β*-alaninate ions link the [Pt(*β*-Ala)_2_I_2_] fragments with K^+^ ions, thus forming polymer layers in the structure (Figure 5). The environment of the K ion includes three O atoms of the carboxylate groups, two O atoms of the water molecules (*d*_K–O_ = 2.780(4) − 2.863(5) Å), and two I ions (*d*_K–I_ = 3.797(1) and 4.285(2) Å), because of which the layers are linked into a framework.

## 4. CONCLUSION

First, the techniques have been developed of the synthesis of bisaminoacid complexes Pt(II) and Pd(II): (1) the interaction of K_2_[PtCl_4_] or Na_2_[PdCl_4_] with *β*-alanine resulted in the *trans*-isomers only; (2) the synthesis of the *cis*-[Pt(*β*-Ala)_2_] can be done by the interaction of K_2_[PtI_4_] with *β*-alanine; (3) for the synthesis of the *cis*-[Pd(*β*-Ala)_2_], we have used the kinetic and thermodynamic data for the *trans*-*cis* isomerization of the Pd(II) bischelates with *α*-amino acid. Second, the investigation of the NMR, IR spectral, and crystal structures have been reported for the individual isomers: the *trans*-[M(*β*-AlaH)_2_Cl_2_] and the *cis*-[M(*β*-Ala)_2_] (M = Pt, Pd).

## Figures and Tables

**Scheme 2:**
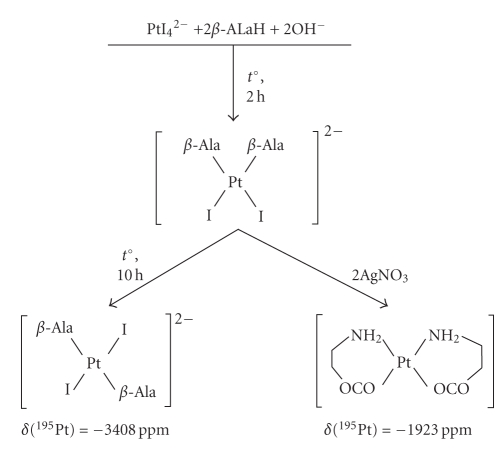
Sequence of reactions of K_2_[PtI_4_] with *β*-alanine.

**Table 1:** NMR spectral data for *β*-AlaH and the Pt(II) and Pd(II) complexes with *β*-AlaH.

Complex (solvent)	^195^Pt NMR, *δ*, ppm	^1^H NMR, *δ*, ppm			^13^C NMR, *δ*, ppm			
		NH_2_	CH_2_–NH_2_	CH_2_–COO	COOH	COO^−^	CH_2_–NH_2_	CH_2_–COO
NH_3_^+^–(CH_2_)_2_–COO^−^ (D_2_O)	—	—	3,10	2,48	179,1		37,30	34,29
*Trans*-[Pt(*β*-AlaH)_2_Cl_2_] (acetone-d_6_)	−2248	* * 4,29^2^J_H–Pt_ = 50 Hz	2,98	2,91	172,9		41,90	33,36
*Trans*-[Pd(*β*-AlaH)_2_Cl_2_] (acetone-d_6_)	—	3,45	2,93	2,87	172,7		40,57	34,01
*Trans*-[Pt(*β*-Ala)_2_] (D_2_O)	−1708	—	2,81	2,44	—		—	—
*Cis*-[Pt(*β*-Ala)_2_] (D_2_O)	−1930	* * 5,17*^2^J_H–Pt_ = 60 Hz	* * 2,76^2^J_H–Pt_ = 45 Hz	2,43	180,2		* *41,87^2^J_C–Pt_ = 30 Hz	* *36,36^3^J_C–Pt_ = 22 Hz
*Trans*-[Pd(*β*-Ala)_2_] (D_2_O)	—	—	2,49	2,38	181,7		38,48	37,82
*Cis*-[Pd(*β*-Ala)_2_] (D_2_O)	—	4,34*	2,59	2,46	180,6		39,86	37,04

* Residual NH_2_ protons.

**Table 2:** IR spectral data for the Pt(II) & Pd(II) complexes with *β*-Alanine.

Complex	$\tilde{\nu }$ (NH), cm^−1^		$\tilde{\nu }$ (C = O), cm^−1^
*Trans*-[Pt(*β*-AlaH)_2_Cl_2_]	3285	3254	1707
	3208	3127	
*Trans*-[Pd(*β*-AlaH)_2_Cl_2_]	3319	3260	1711
		3128	
*Trans*-[Pt(*β*-Ala)_2_]	3268	3215	1617
	3184	3090	
*Trans*-[Pd(*β*-Ala)_2_]	3225	3038	1628
*Cis-*[Pt(*β*-Ala)_2_]	3235	3047	1640
*Cis*-[Pd(*β*-Ala)_2_]	3233	3080	1634
		3037	
*Trans*-K_2_[Pt(*β*-Ala)_2_I_2_]	3246	3190	1602
		3069	

**Table 3:** Selected stereochemical data for the studied compounds.

	*Trans*-[Pd(*β*-Ala)_2_]	*Trans*-[Pt(*β*-Ala)_2_]	*Cis*-[Pt(*β*-Ala)_2_]	K_2_[Pt(*β*-Ala)_2_I_2_]^•^2H_2_O
Pt–O	2.004(2)* *2.004(2)	1.997(5)* *1.996(6)	1.994(7)* *2.033(7)	—
			2.019(7)* *2.014(8)	
Pt–N	2.026(2)* *2.026(2)	2.031(7)* *2.026(8)	1.979(9)* *2.015(8)	2.047(5)
			1.986(9)* *2.009(7)	
Pt–Hal	—	—	—	2.5902(5)
K–I	—	—	—	3.797(1)
				4.285(2)
K–O	—	—	—	2.780(4)* *2.820(5)
				2.808(5)* *2.863(5)
				2.851(4)
∠ NPtO	94.35(8)	94.0(3)	95.1(3)* *95.0(3)	—
			96.1(3)* *94.9(3)	
C–O	1.291(3)* *1.232(2)	1.290(11)* *1.235(11)	1.291(15)* *1.188(15)	1.249(7)* *1.255(7)
			1.308(13)* *1.186(14)	
		1.281(10)* *1.221(11)	1.259(14)* *1.252(14)	
			1.317(13)* *1.219(14)	
C–N	1.463(3)	1.486(11)* *1.461(14)	1.525(15)* *1.461(15)	1.489(8)
			1.539(14)* *1.468(14)	

## References

[1] Rosenberg B (1971). Some biological effects of platinum compounds. *Platinum Metals Review*.

[2] Volstein LM (1975). *Russian Journal of Coordination Chemistry 
*.

[3] Iakovidis A, Hadjiliadis N (1994). Complex compounds of platinum (II) and (IV) with amino acids, peptides and their derivatives. *Coordination Chemistry Reviews*.

[4] Krylova LF, Pavlushko TA (2003). Diastereomers of *trans* isomers of Pt(II) complexes with alanine and phenylalanine. *Russian Journal of Inorganic Chemistry*.

[5] Krylova LF, Golovin AV (2000). NMR studies of transformations of aminoacid complexes of Pt(II) and Pd(II) in solution. 2. Bisalaninates. *Journal of Structural Chemistry*.

[6] Chernova NN, Shahova LP, Kukushkin YuN (1976). *Russian Journal of Inorganic Chemistry 
*.

[7] Sullivan EA (1979). Conformational dissymmetry. Circular dichroism of amino acid and peptide complexes. *Canadian Journal of Chemistry*.

[8] Taubald U, Nagel U, Beek W (1984). Dichloropalladium(II)-komplexe mit *α*-aminosäuren, *α*-aminosäureestern, dipeptiden und dipeptidestern. *Chemische Berichte*.

[9] Volstein LM, Mogilevkina MF (1955). *Russ. Dokl. AN SSSR *.

[10] Leonova TN, Muraveiskaya GS, Shchelokov RN (1979). *Russian Journal of Inorganic Chemistry *.

[11] Cavoli P, Graziani R, Casellato U, Uguagliati P (1986). Mechanism of reaction of zeise's salt with *β*-alanine. Crystal and molecular structure of *trans*-(N,olefin)[Pt(C_2_H_4_)(*β*-alaninato)Cl]. *Inorganica Chimica Acta*.

[12] Krylova LF, Pavlushko TA (2001). Isomeric heteroligand platinum(II) complexes with amino acids of the glycine series. *Russian Journal of Inorganic Chemistry*.

[13] Volstein LM, Krylova LF, Belyaev AV (1973). *Russian Journal of Inorganic Chemistry 
*.

[14] Krylova LF, Golovin AV (2000). NMR studies of transformations of Pt(II) and Pd(II) 
complexes with amino acids in solution. 1. Glycine complexes. *Journal of Structural Chemistry*.

[15] Krylova LF, Kuprov IS (2003). Stereoisomeric Pd(II) complexes with valine. *Russian Journal of Inorganic Chemistry*.

[16] Krylova LF, Matveeva LM (2005). Identification of Pt(II) and Pd(II) stereoisomeric complexes with aminobutyric 
acid by NMR and IR spectroscopy. *Journal of Structural Chemistry*.

[17] Günther H (1979). *NMR Spectroscopy: An Introduction*.

[18] Krylova LF, Kuprov IS (2001). Stereoisomeric Pt(II) complexes with valine. *Russian Journal of Inorganic Chemistry*.

[19] Nakamoto K (1986). *Infrared and Raman Spectra of Inorganic and Coordination Compounds*.

